# Supplementary material for: Association between oral microbiome and seven types of cancers in East Asian population: a two-sample Mendelian randomization analysis
Source: Front Mol Biosci. 2023 Nov 21;10:1327893. doi: 10.3389/fmolb.2023.1327893 (PMC10702768; doi:10.3389/fmolb.2023.1327893)
Supplement: Supplementary file 1 [file DataSheet1.ZIP › Supplementary Materials/MR plots of saliva/saliva═╝/Pancreatic cancer/pheno.3386_to_pancreatic cancer_leave_one_out.pdf]

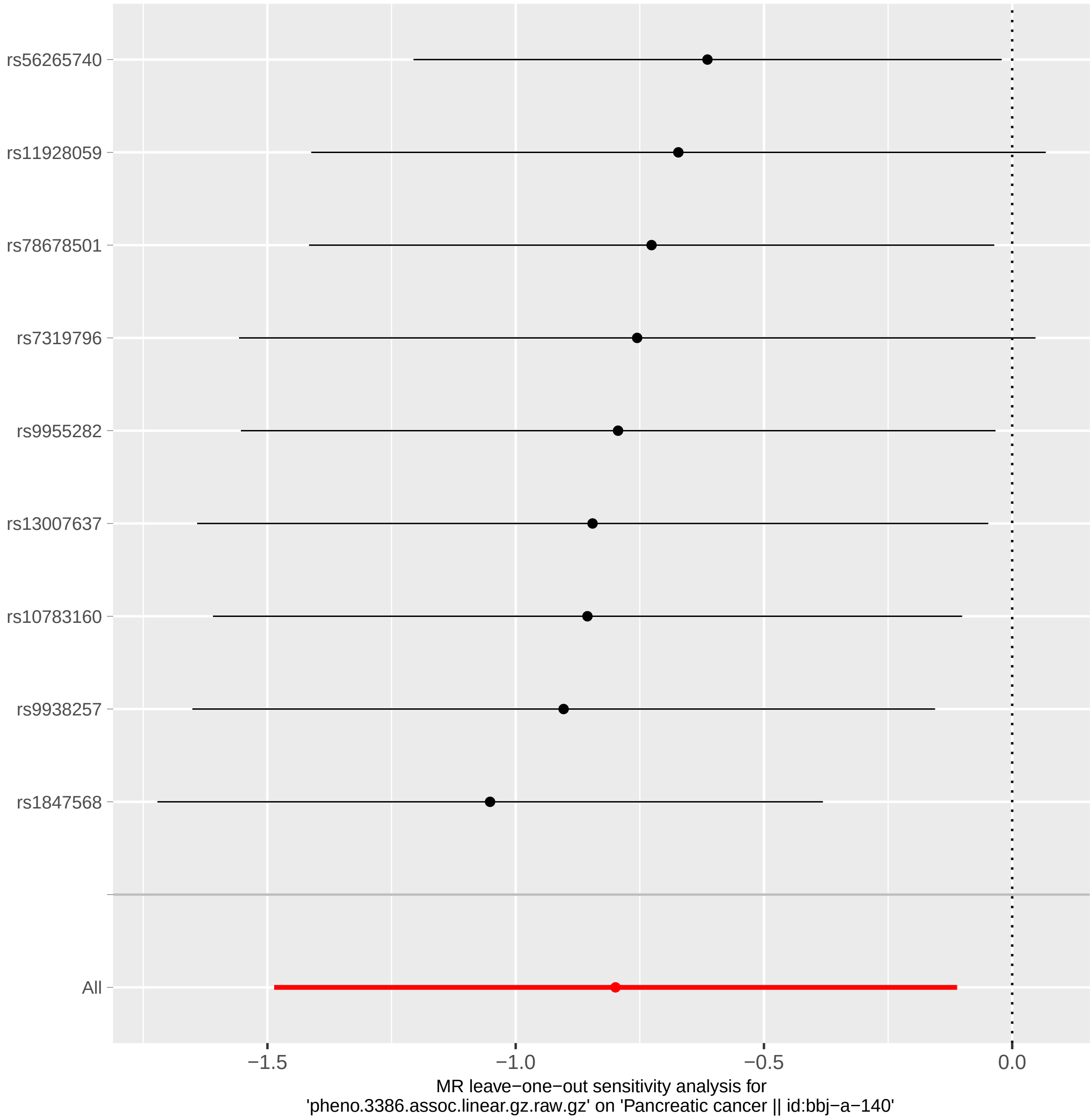

rs56265740
rs11928059
rs78678501
rs7319796
rs9955282
rs13007637
rs10783160
rs9938257
rs1847568
All
−1.5
−1.0
−0.5
0.0
MR leave−one−out sensitivity analysis for
'pheno.3386.assoc.linear.gz.raw.gz' on 'Pancreatic cancer || id:bbj−a−140'
